# The Cladistic Basis for the Phylogenetic Diversity (PD) Measure Links Evolutionary Features to Environmental Gradients and Supports Broad Applications of Microbial Ecology’s “Phylogenetic Beta Diversity” Framework

**DOI:** 10.3390/ijms10114723

**Published:** 2009-11-03

**Authors:** Daniel P. Faith, Catherine A. Lozupone, David Nipperess, Rob Knight

**Affiliations:** 1 The Australian Museum, 6 College St., Sydney, NSW, 2010, Australia; 2 Department of Chemistry and Biochemistry, University of Colorado, Boulder, CO 80309, USA; E-Mails: catherine.lozupone@colorado.edu (C.A.L.); Rob.Knight@colorado.edu (R.K.); 3 Department of Biological Sciences, Macquarie University, NSW, 2109, Australia; E-Mail: dnippere@bio.mq.edu.au (D.N.)

**Keywords:** bacteria, UniFrac, feature diversity, ordination, PD calculus, evolutionary features, GEO BON, climate change

## Abstract

The PD measure of phylogenetic diversity interprets branch lengths cladistically to make inferences about feature diversity. PD calculations extend conventional species-level ecological indices to the features level. The “phylogenetic beta diversity” framework developed by microbial ecologists calculates PD-dissimilarities between community localities. Interpretation of these PD-dissimilarities at the feature level explains the framework’s success in producing ordinations revealing environmental gradients. An example gradients space using PD-dissimilarities illustrates how evolutionary features form unimodal response patterns to gradients. This features model supports new application of existing species-level methods that are robust to unimodal responses, plus novel applications relating to climate change, commercial products discovery, and community assembly.

## 1. Introduction

Cladistic analyses are based on a well-known assumption about the relationship between features (character states) and phylogenetic pattern: shared ancestry can account for shared features among taxa. That fundamental model is a basis for inference of phylogeny from observed character data for a set of taxa. Cladistic parsimony methods prefer trees that maximize this particular explanation of character variation; most-parsimonious trees equivalently minimize the sum of branch “lengths” that count character state changes. This inference of phylogenetic trees from characters is well-established, but this same cladistic model also means that we can “work backwards”. Starting with a phylogeny and some estimate of branch lengths, we can make inferences about the relative number of features arising in a given branch, and the relative feature diversity of a given set of taxa. The well-known PD (phylogenetic diversity) measure [1,2] in this way explicitly links the cladistic model to measures of feature diversity or PD. The PD measure is based on the assumption that shared ancestry indicates shared features. Faith [1,2] also has discussed the robustness of PD to convergent evolution and other departures from this model.

Interpretation of PD as counting-up features for different sets of taxa means that we can interpret various PD calculations as in effect operating at the features level. A family of PD measures now extends conventional species-level measures and indices to the features level [3]. For example, “PD-endemism” [4] uses phylogenetic branch lengths and geographic distribution information to indicate the extent to which evolutionary features are restricted to a given region.

In this paper, we extend PD’s cladistic interpretation of phylogenetic branches as a proxy for evolutionary features. Microbial ecologists have developed a PD-based dissimilarity measure based on calculations using branch lengths. We will show how interpretation of these PD-dissimilarities at the level of features provides a model that justifies the microbial approach and points to new applications.

## 2. Microbial Ecology and PD-Dissimilarities

Microbial ecologists demonstrate well that necessity is the mother of invention. In the absence of conventional species-level data, they have developed innovative ways to study patterns of microbial diversity—for example, using only microbial phylogenetic patterns inferred from molecular sequence data. Pioneering work includes a useful approach that microbial ecologists call “phylogenetic beta diversity” [5–8]. “Beta diversity” is an ecological term conventionally applied at the species level. It refers to measures of the turnover in species composition among sample sites [9]. When two sites are compared, beta diversity can be indicated by a measure of their dissimilarity in species composition. Microbial ecologists have calculated these dissimilarities using phylogenetic patterns. The phylogenetic dissimilarities in their phylogenetic beta diversity studies are based explicitly on PD calculations using branch lengths [5,8]. Thus, these dissimilarities (see also [10]) extend early versions of PD-dissimilarity, as in the calculation of the total branch length found in one locality but not in another (“PD-complementarity” [1,4]).

**Figure 1.**
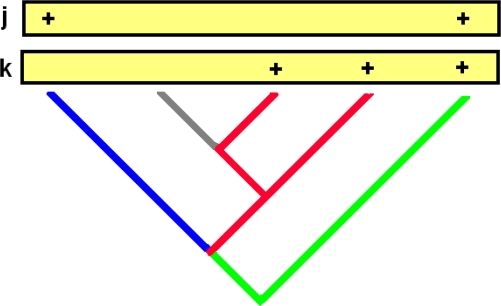
A portion of a hypothetical phylogenetic tree showing 5 taxa. There are two localities, **j** and **k**, indicated by rectangles at the top of the tree. For each locality, ‘+’ designates presence of a taxon. Blue branches are only represented in **j**; red branches are only represented in **k**; green branches are represented in both; the gray branch in neither. These designations can be used to calculate PD-dissimilarities. For details, see text following the figure. The PD-complementarity [1,4] of **j**, given **k**, counts the gain in branch representation (the blue branch lengths), and the PD-complementarity of **k**, given **j**, similarly counts the red branch lengths. A PD-dissimilarity, analogous to the presence-absence version of Bray-Curtis dissimilarity [10], counts the branches represented in **j**, not **k** (length of blue branches), plus the branches represented in **k**, not **j** (length of red branches), divided by the sum of the total branch length represented in each (length of blue plus length of green branches, plus length of red plus length of green branches). Both Lozupone and Knight [8] and Ferrier *et al.* [10] have recognised a family of PD-dissimilarities that vary in how these matched and miss-matched branch counts are combined. The microbial study of Bryant *et al.* [11] applied a measure equivalent to the Bray-Curtis type PD-dissimilarity defined in [10]. UniFrac [5] applications have calculated the number of features in **j**, not **k** (length of blue branches), plus the number of features in **k**, not **j** (length of red branches), divided by the sum of the total number of features found in either (length of blue plus length of red, plus length of shared green branches). This is a phylogenetic analogue of one of the Bray-Curtis type measures found to be most robust by Faith *et al*. [12]. Note that for the UniFrac and other PD-dissimilarities, the green branch on the left side is recorded as shared by **j** and **k**, even though the two sites do not share any of the species descendent from that branch.

PD-dissimilarities among localities (or between sets of localities) are calculated using phylogenetic tree branches, producing measures analogous to Bray-Curtis and other species-level dissimilarities [11] (Figure 1). These phylogenetic dissimilarities have been used in ordinations to reveal key environmental gradients, as in the discovery that salinity helps explain global microbial phylogenetic beta diversity patterns [6]. A software toolbox, UniFrac [5], has extended phylogenetic beta diversity applications, supporting ordination and other dissimilarities-based analyses, including regressions, clustering, randomization tests, and various other diagnostics [5,8].

UniFrac has been applied broadly in microbial biology, with more than 80 papers using it to compare microbial assemblages, as of this writing. These include bacterial [13,14], archaeal [15], eukaryotic [16,17], and viral [18,19] assemblages important for understanding human health and disease [20–24], bioremediation [25], and basic ecology and evolution [11,26,27]. The ordinations have revealed a diversity of environmental gradients, such as a seasonality gradient for house dust microbial communities [14], a diet gradient for mammal faecal microbial communities [28], and a depth gradient for layers in a microbial mat [13].

The microbial studies have laid the foundations for phylogenetic beta diversity studies by demonstrating repeatedly that PD-dissimilarities recover gradients, and may accomplish this better than non-phylogenetic analyses [29]. In addition, microbial ecologists have also tested the sensitivity of phylogenetic beta diversity patterns to various factors. For example, phylogenetic beta diversity appears to be robust to imperfections in phylogenetic tree construction. In one example, when a comparison of bacteria from hot spring sediments collected across temperature and chemical gradients was performed using trees generated with several of the most popular methods of phylogenetic tree constructions, similar ordination patterns were observed despite considerable differences in both topology and branch lengths in the trees [8]. The UniFrac software [5] also provides a jackknifing technique in order to investigate sensitivity of results (*e.g.*, clusters) to sampling intensity as reflected in variation in number of sampled taxa. Microbial ecologists also have demonstrated the flexibility of the UniFrac approach for investigating key processes, including detection of horizontal gene transfer, where phylogenetically disparate organisms in similar habitats converge in gene content [30].

The range of successful studies suggests that these methods could be the basis for broader applications of phylogenetic beta diversity in evolutionary ecology. This is a timely issue given recent promotion, by Graham and Fine [31], of “phylogenetic beta diversity” as an exciting new frontier, but without reference to the established “phylogenetic beta diversity” applications of microbial ecology. Similarly, other recent work [32], while acknowledging a range of possible approaches falling under the term, “phylogenetic beta diversity”, do not cite the origin of this term in the earlier study of Lozupone *et al*. [7] (see also [5]).

Graham and Fine did refer to possible phylogenetic beta diversity measures: “similarity between communities, such as the Jaccard or Sorenson’s index also could be explored…this metric could be calculated as the total branch length covered by shared species relative to the total branch length covered by all species in both communities.” However, this suggestion does not capture the insight, embodied in the established PD-complementarity and PD-dissimilarity measures, that two sites can share (represent the same) branches even without sharing any species (Figure 1; see also [33]).

The recent new-frontiers perspective therefore contrasts with the well-established use of specific PD-dissimilarities in the microbial phylogenetic beta diversity studies. Indeed, given that PD-dissimilarities have been trialed successfully in microbial ecology, it is tempting to simply recommend that approach for general use. However, there are alternative ways to calculate some kind of phylogenetic distance between samples or communities. For example, Webb *et al*. [34] describe “phylogenetic ordinations” that measure phylogenetic distance between samples by calculating the average branch-length (phylogenetic path-length) distance, over all possible pairings produced by taking one taxon from each sample.

While acknowledging that there are a range of approaches, we will argue that PD-dissimilarities have desirable properties as a general foundation for considering environmental gradients and phylogenetic beta diversity. We will support this claim by providing a so-far missing ingredient in phylogenetic beta diversity studies—a general model linking specific PD-dissimilarities to environmental gradients, and to corresponding robust analysis methods. Our rationale is that: (1) based on the underlying cladistic model, PD-dissimilarities approximate the quantities we would get if we could directly calculate dissimilarities using evolutionary features, (2) evolutionary features in turn can be linked, by a simple “unimodal response” model, to environmental gradients, and (3) PD-dissimilarities relate to unimodal response of features in the same way that conventional robust dissimilarities relate to unimodal response of species - allowing phylogenetic application of the existing robust analysis methods for inferring environmental gradients. This model not only will justify the general application of the microbial phylogenetic beta diversity approach, but also will provide a robust foundation for new applications such as product discovery and monitoring for human impacts.

## 3. The Unimodal Response Model for Evolutionary Features

There is a well-established link at the species level between dissimilarities among localities and gradients or ordination space. Under a model of general unimodal species’ “response” to gradients, Bray-Curtis (and related) dissimilarities are approximately monotonically related to distances in the underlying gradients space [12]. Ordination methods, such as Hybrid Multidimensional Scaling, have built on this relationship to provide robust methods for inference of environmental gradients [12,35].

**Figure 2.**
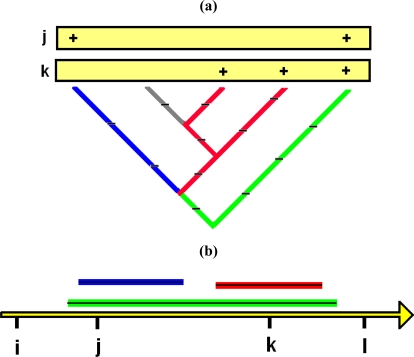
A hypothetical phylogenetic tree, and the distribution of taxa in two localities, illustrates the link to distributions of features along environmental gradients. For details, see text following the figure. (a) The portion of a hypothetical phylogenetic tree showing 5 taxa, and two localities, **j** and **k**, from Figure 1. We now interpret the branch lengths as counting derivation of evolutionary features, according to the PD model [1,2], and we graphically indicate these features with tick marks in accord with the original PD studies [1,2]. The blue branches indicate features only represented in **j**; red branches indicate features only represented in **k**; green branches indicate features represented in both; the gray branch indicates features in neither. The PD-dissimilarity, analogous to the presence-absence version of Bray-Curtis dissimilarity [10], counts the number of features in **j**, not **k** (length of blue branches) plus the number of features in **k**, not **j** (length of red branches), divided by the sum of the total number of features found in each (length of blue plus length of green branches, plus length of red plus length of green branches). (b) Because each branch represents new evolutionary features arising along that branch, showing the set of sites (along a hypothetical underlying environmental gradient) that represent a given branch also shows the set of sites that have the corresponding features. This gradients diagram illustrates unimodal responses for such evolutionary features. The hollow-line arrow is a hypothetical gradient with localities **i**, **j**, **k**, and **l**. Under the unimodal response model, the features in both **j** and **k** (green branches in (a)) form the green line segment, with a black line graphically indicating the identical distribution of the corresponding features along the gradient. Blue and red branches from (a) similarly correspond to line segments along the gradient, representing the distribution of the corresponding features along the gradient. Line segments are stacked above the gradient in order to show individual coloured distribution extents. These line segments reflect the simple presence-absence case of unimodality (“binary” case [12], see also [36]). Abundance information would transform the segment into a single-peaked curve, where some point along the segment corresponds to maximum abundance. Under the assumptions of the unimodal model, Bray-Curtis type PD-dissimilarities (including that in UniFrac), applied to observations on the presence/abundance of features (branches) in localities, can be used in robust ordination methods to recover key gradients.

This same robust framework may extend from species to phylogeny. While PD-dissimilarities operate by counting-up shared versus not-shared branch lengths, interpretation of these calculations at the level of evolutionary features helps establish a link between observed communities and underlying environmental gradients. Based on PD’s cladistic model, PD-dissimilarities, which were interpreted as calculations using branch lengths in Figure 1, also can be interpreted as if we had calculated dissimilarities directly on the evolutionary features represented by branches (Figure 2a). This interpretation suggests that we can borrow from the established rationale for linking species-level dissimilarities to gradients: a Bray-Curtis type PD-dissimilarity will provide robust inference of gradients—when a unimodal response at the level of features is present. This means that evolutionary features, including those represented by non-terminal phylogenetic branches, would have general unimodal responses to gradients (Figure 2b). Such a unimodal model for features is plausible (e.g., [36,37]), but has not been evaluated for any of the microbial studies where PD-dissimilarities have recovered environmental gradients.

We re-examined the gradient space, derived using Bray-Curtis type PD-dissimilarities, from Rintala *et al.*’s recent study [14] of house-dust microbial communities. This UniFrac analysis had recovered underlying environmental gradients, including those related to different buildings and to different seasons. We recorded the taxa—and therefore the phylogenetic branches—represented by (“present in”) each sample locality, and then examined the positions in gradients space of all those sample localities representing any given branch. We assessed unimodal responses by recording as “unimodal” each case where a simple convex shape could enclose all those sample localities representing a given branch (and so the features represented by the branches), and not include any other localities.

**Figure 3.**
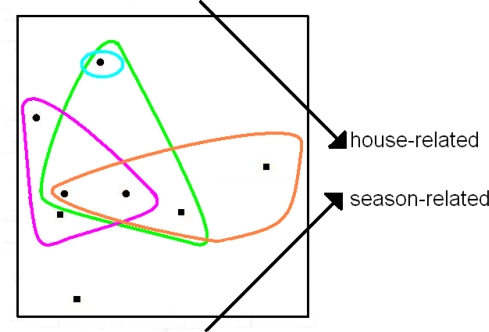
A re-drawing of the gradient space from Rintala *et al.* [14] for microbial communities in house dust. Dots versus squares correspond to samples from two different buildings (for details of sampling see [14]). Arrows at the right side indicate major gradients revealed by the ordination. A sample locality represents the branch corresponding to a given family if the locality has one or more descendants of that branch. For details, see text following the figure. Unimodal response was recorded when a shape can enclose all sample localities representing a given branch, and not include any other localities. The coloured shapes illustrate unimodal response patterns for the non-terminal phylogenetic branches represented by identified families in the phylogenetic tree used in their study (see Figure 2 in [14]). As for Figure 2b, the diagram indicates binary unimodal response. Green shape—Acidaminococcaceae, Purple shape—Aerococcaceae, Orange shape—Enterobacteriaceae, Blue shape—Acetobacteraceae. For Rintala *et al.*’s full 3-dimensional gradients space, of the 56 identified families (representing phylogenetic branches) all but 3 form a clear unimodal response. For the 8 higher level taxa identified by Rintala *et al.*, all but 1 forms unimodal response by our criterion. However, at this higher taxonomic level, many of the taxa appear at all sites. Complete unimodality assessments are available from the corresponding author of this paper.

We found unimodal response patterns in the gradient space (Figure 3), even for the non-terminal phylogenetic branches (corresponding to 56 named families; see Figure 2 in [14]). This strong pattern linking features to gradients provides some first evidence supporting this model and methods combination as a robust framework for phylogenetic beta diversity. It suggests that the general success of microbial phylogenetic beta diversity studies can be attributed to the use of PD-dissimilarities that are robust to general unimodal response of evolutionary features to gradients. This robustness means that the PD-dissimilarities, like robust species-level dissimilarities [12], will have an approximately monotonic relationship to distances in the underlying gradients space, providing a pathway for inferring gradients from observed phylogenetic community differences. We anticipate that future phylogenetic beta diversity studies, using UniFrac and other PD-dissimilarities, will reveal key environmental gradients and further document unimodal response patterns for the features represented by phylogenetic branches.

This result does raise the question as to whether conventional species-level analysis—which also can reflect response of evolutionary features to gradients - might provide enough information on its own to recover gradients. We suggest that use of phylogenetic pattern generally will improve recovery of gradients, based on unimodal response patterns for the features represented by deeper branches. A reason is that the successful recovery of gradients is enhanced when there are unimodal responses spread over the length of the underlying gradient, and the deeper branches help provide this. This contribution of deeper branches may explain cases [11,29] where phylogenetic level analysis differs from species level analysis (see also [38]). This conjecture calls for investigation through other case studies.

Our interpretation of phylogenetic beta diversity and PD-dissimilarities at the feature-level has taken advantage of the fundamental cladistic model in which “shared ancestry accounts for shared features”. This in turn has helped to document a companion feature-level model for ecology where “shared environment accounts for shared features” (see also [36]).

## 4. Some Implications for Choice of Methods Used to Explore Patterns of Diversity

Different disciplines, including microbial ecology, tend to favour different multivariate methods to study community patterns [39]. The unimodal response model can help to identify those methods that deserve more general application. Certainly, any preferred method must be robust to departures from strict unimodality, given that the unimodal model does not demand that all feature responses are perfectly unimodal (for discussion, see [12,40]). This argument supports the use of robust ordination methods such as Hybrid Multidimensional Scaling [12]. Robustness is also an issue for cluster analysis methods. Clustering of PD-dissimilarities from UniFrac has been used to reveal environmental factors, as in the finding that different soil types are reflected in clusters of bacterial communities [41]. Perhaps such clustering will be most successful when the clustering method is one that implicitly views the clusters in continuous gradients space, and is compatible with an underlying unimodal model (e.g., [42]).

Central to all these robust approaches is the expectation that Bray-Curtis type PD-dissimilarities (Figures 1 and 2) will have a relatively tight monotonic relationship with ordination/environmental distances, paralleling the relationship already described at the species level [12]. This monotonic relationship, under the unimodal response model, explains why we can expect well-defined patterns of “distance decay” (compare to [11,43]), in which dissimilarity increases with environmental or geographic distance.

Koleff *et al.* [44] discuss a wide range of beta diversity measures at the species level for presence-absence data. All of those measures have direct phylogenetic beta diversity counter-parts, through PD calculations based on the counting of matches and mismatches as in Figure 1. Koleff *et al.* linked properties of beta diversity measures to gradients. Koleff *et al.* sought dissimilarity measures such that the beta diversity (*e.g.*, dissimilarity) between two localities far away on a gradient would be the sum of values between localities along the way. This goal appears to be unrealistic under a model of unimodal response. The monotonic relationship between dissimilarity and gradient distance that results under the unimodal response model [12] means that the overall value will be less than this sum of individual values. Thus, the criterion proposed by Koleff *et al.* could lead to a preference for a less robust dissimilarity measure. If beta diversity is to have some strong link to inference of gradients, then Bray-Curtis type measures [12] have special status, both for species and phylogenetic dissimilarity calculations.

The monotonic dissimilarity/distance relationship also justifies the use of Bray-Curtis type PD-dissimilarities in regressions of compositional dissimilarities on environmental distances. Generalized dissimilarity modeling (GDM [10]) is an extension of matrix regression that allows for a monotonic, curvilinear, relationship between increasing environmental distance and compositional dissimilarity, and allows also for variation in the rate of compositional turnover at different positions along environmental gradients. GDM has been suggested as a useful approach for predicting PD-dissimilarities (*e.g.*, for predicting the dissimilarity between two un-sampled localities [10]). The unimodal response model supports an expectation that GDM will successfully predict Bray-Curtis type PD-dissimilarities.

## 5. Some Implications for Methods Used to Explore Processes Driving Community Composition

A good fit between PD-dissimilarities and ordination/environmental distances (*e.g.*, as indicated by a significantly low stress value in a multidimensional scaling) will suggest that sites that share features or branches also have similar habitats. Thus, the unimodal model combined with robust ordination methods provides a framework for exploring “habitat filtering” (*e.g.*, [11,45]), where the distribution among sites of features/branches is largely determined by limiting environmental or habitat factors (Figure 3).

The other major factor in determining community composition, competition between species, would appear to be less clearly addressed by the gradient-space approach. An hypothesis of competition as a determinant might be supported by finding patterns where there is evidence of phylogenetic “repulsion”—in which closely related species are less likely to co-occur in a community [46]. Recent phylogenetic approaches to this problem (*e.g.*, [45,47,48]) interpret phylogenetic over-dispersion in a community (where closely related species do not co-occur) as evidence of competition. Some studies have attempted to first use environmental factors to explain community composition, and then have asked whether there remains evidence for over-dispersion [48].

This kind of approach might be enhanced by phylogenetic beta diversity analyses. The unimodal response of features in gradients space provides a natural way to explore evidence for competition, after taking into account environmental filtering. For example, suppose that the expected correspondence between PD-dissimilarity and environmental distance is defined by the GDM regression method [10], and then the predicted dissimilarities based on GDM-scaled environmental distances are used to derive the ordination. Because the environmental distances determine location of sites in ordination space, two communities with very similar environmental values may be forced together even if they have some degree of PD-dissimilarity. This result would correspond to some high residual dissimilarity values in the GDM regression (for examples of GDM residuals, see [10]). These residuals may reflect cases where closely related lineages do not occur in the same site, but do occur in separate sites that are similar environmentally.

**Figure 4.**
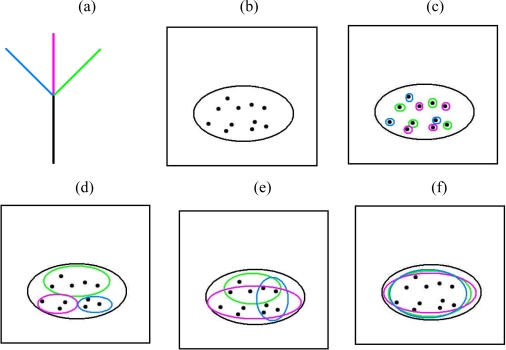
An example based on a simple phylogenetic tree and a gradients space illustrates inferences about competition and habitat filtering. For details, see text following the figure. (a) A portion of a hypothetical phylogenetic tree used to calculate PD-dissimilarities among sites. The tree portion shows an ancestral branch (black) and three descendent branches (blue, green, purple), leading to three descendent species. (b) A hypothetical gradient space in which the expected correspondence between the PD-dissimilarities and environmental distances is defined by the GDM regression method, and then the predicted dissimilarities based on GDM-scaled environmental distances are used to derive an ordination [10]. Sample sites are shown as black dots. (c) The black oval outlines the distribution among sites of the ancestral black branch in (a). The distribution among sites of the three branches from (a) are shown as ovals. As in Figure 2, the distribution among sites of branches indicates the distribution of corresponding features. The branches corresponding to the 3 species do not co-occur, and the differences in distribution among sample sites are not explained by habitat filtering. This pattern supports an hypothesis that competition has been a determinant of community composition. (d) Based on coloured ovals defined as in (a), the pattern shows that the species partition the gradient space and do not co-occur in communities. However, competition is not strongly implicated, as habitat filtering can explain the species distributions in space. (e) Here, species partly overlap and also to some degree show habitat filtering. Competition is not implicated. (f) The species co-occur in the same communities.

Such initial evidence for competition would be strengthened by examining individual clades, corresponding to non-terminal branches, and their descendent lineages, in the gradients space. While model residuals reflect the entire phylogeny, an individual clade might provide evidence for competition within that clade. Figure 4 illustrates possible patterns that might be observed in the ordination space based on the distribution among sites of the clade and its descendent lineages. Scenario (c) suggests that competition could have been a determinant of community composition. Here, different lineages/features, within the larger clade, do not occur in the same sites/communities.

To illustrate this approach, we re-examined the Rintala *et al*. study [14] and searched for patterns illustrating these scenarios. Figure 3 showed distributions for Acidaminococcaceae (Purple) and Aerococcaceae (Green), two of 12 listed families [14] within the phylum Firmicutes. All sample sites have at least one family from this phylum. Further, the different families, including Acidaminococcaceae and Aerococcaceae (Figure 3), often co-occur in the same sites [14]. The pattern in gradients space for Firmicutes therefore resembles Figure 4e. The combination of unimodal response patterns for the families, and the occurrence of these families in many of the same sites, does not provide any evidence for competition at this level.

When a scenario similar to Figure 4c is found, the evidence for competition will be stronger if:

1. Co-occurrence in sites of the lineages within that larger clade is significantly low, as indicated by an appropriate randomization test.
2. The unimodal response pattern for these lineages (clumping of those sites having the lineage) is absent, suggesting that distribution of the lineage among sites is not simply determined by further specialization within environmental space.
3. The GDM residuals for specific pairs of sample sites (where both sites contain members of the larger clade) correspond to higher PD–dissimilarity values than expected.

These forms of evidence suggest that unimodal response patterns in gradients space - including those for deeper phylogenetic branches - provide a unique opportunity to explore the relative roles of habitat and competition determinants of community composition. These approaches could add to the expanding toolbox of methods linking phylogeny and community assembly (for review, see [49]).

## 6. Extending Phylogenetic Beta Diversity Applications

The unimodal model supports inference of a gradients space, but the model also allows us to reverse the inference process—using the gradients space to make predictions about features. A useful property of the unimodal model for phylogenetic beta diversity is that it informs us about patterns of turnover in features as we move across the gradients space. We expect to find communities with many different features if we move to a community far away in the space. Suppose we have a new prospective sample locality which has not yet been sampled, but has a known position in gradient space based on its environmental values (*e.g.*, using scaled environmental distances from GDM). We can predict how many new features (relative to all the existing sample localities) would be contributed by the new sample locality. In conventional “survey gap analysis” [10], a method, “ED” (“environmental diversity” [50,51]) uses p-median and related criteria to assess how big a gap would be created in gradients space if a site were removed. This gap-size, or ED-complementarity value, indicates the number of new species represented by a new sample site, under an assumption of species’ unimodal responses [for an example of ED calculations, see [51]). We can use the same ED method to predict *feature* gains, assuming unimodal response at the features level. Thus, the unimodal model justifies the application of the ED approach for survey design, to evaluate localities that may offer not-yet sampled microbial diversity (more generally, any phylogenetic diversity).

### 6.1. The Search for New Commercial Products

Phylogenetic survey gap analyses may greatly extend the use of phylogenies to search for new commercial products. The PD measure is already well-established as a way to sample phylogeny in order to maximize representation of useful feature diversity. For example, Forest *et al.* [52] illustrated how choosing a representative set of plant taxa based on PD would maximize the probability of having representatives of identified classes of human-use. Pacharawongsakda *et al.* [53] recently established a microbial sampling program based on PD assessments, with the goal of finding new commercial products.

PD is an effective strategy when we are working with specific phylogenetic trees, but PD-dissimilarities and phylogenetic beta diversity may extend these applications. The inference of a gradients space, followed by application of the ED survey gap methods, could provide needed phylogenetic and geographic extrapolation. First, ED may predict which communities/places offer new features, not just for the phylogeny used to derive the gradients space, but for other phylogenies as well—assuming these same environmental gradients are relevant to the other taxonomic groups. Second, because ED builds on GDM and other methods to place new, unsampled, localities in gradients space, it is possible to judge new sites’ likely contribution of new features and products. PD on its own cannot provide these broader predictions.

### 6.2. Monitoring Human Impacts

We have described how the general unimodal response pattern of features in gradients space allows inferences about the expected phylogenetic community composition at new sample sites that have known environmental values, but unknown composition. This kind of prediction of composition at new sites is the basis for an important form of monitoring for human impacts. Programs such as AUSRIVAS (*e.g.*, [54]) use community-level models to link environmental variables to community composition (using species or higher taxonomic levels). This predictive link is established for a “reference” set of relatively pristine localities. These monitoring programs then can evaluate a new locality, making a prediction of the expected community composition, based on the locality’s environmental values. The actual community composition at this new site then is observed and recorded. Human impact may be inferred if the observed and expected communities are very different [54].

A key to effective monitoring of this kind is a strong model that can predict expected community composition using only environmental variables. The phylogenetic unimodal model, and corresponding GDM predictions of PD-dissimilarities, can provide these predictions. This establishes as a basis for monitoring regional impacts on phylogenetic diversity. In the simplest case, we then can compare a new sample site to a single, environmentally similar, reference site. We then can ask how well the new site’s expected PD-dissimilarity to the reference site (based on GDM) corresponds to the observed PD-dissimilarity (calculated after sampling the new site).

**Figure 5.**
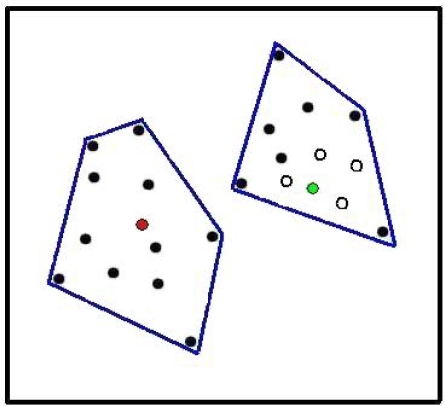
A hypothetical two dimensional gradient space in which solid and hollow dots are reference sample sites. The distribution among sites of two phylogenetic lineages is shown by two corresponding convex hulls (in blue). Solid dots represent reference sites in which the corresponding lineage is present; hollow dots represent reference sites in which the lineage is absent. In each case, a site (red dot, green dot) to be evaluated for impacts has been positioned in the space based on its environmental values.

Better tests might use a gradients space, with localities given by multiple reference sites. For example, consider impacts involving loss of lineages/features and a gradients space derived using phylogenetic beta diversity. For each phylogenetic branch, we calculate a convex hull in the space around all those sites having that linage (Figure 5). We then locate a new site in the gradients space, using GDM. We are interested in those cases where the new site falls within a convex hull for a given phylogenetic branch, but the site does not itself have (any descendants of) that branch/lineage. We can assess the probability that a reference site within that convex-hull-defined region of gradient space would not have that lineage. As an example, Figure 5 illustrates the assessment for two lineages. For the lineage on the left, the absence of that lineage in the red-dot site is assessed as improbable—all reference sites in the convex hull have the lineage. The red-dot site is interpreted as possibly impacted. For the lineage on the right, absence of that lineage in the green-dot site is not assessed as improbable, because the lineage also is absent in some reference sites falling within the convex hull of the lineage (Figure 5). The green-dot site is not interpreted as a site calling for investigation of possible impacts.

Such an assessment can be repeated for all lineages. An hypothesis of impact is well-corroborated if it is highly improbable that such a new site, viewed as an additional reference site, could have had so many missing lineages (for discussion of similar corroboration assessments in monitoring, see [55]).

The inference of impact at a site may be followed up by an assessment of the consequent loss in phylogenetic or feature diversity. The ED calculus allows assessment of the loss in features if a site is “lost” – for example, because of human impacts [50,51]. The phylogenetic beta diversity framework therefore provides a basis for assessment of losses, and may support new initiatives for regional and global scale monitoring of genetic and phylogenetic diversity.

One of the approaches considered as part of a global biodiversity observation network (GEO BON [56]) would use gradients-space models, and methods such as ED, to create a biodiversity “lens” for interpreting remotely-sensed changes in land/water condition. A locality in gradients space may be judged through remote sensing as degraded, and ED estimates the consequent biodiversity loss.

The gradient space examples illustrated in this study (*e.g.*, Figure 3) demonstrate that the unimodal model for phylogenetic beta diversity enables these biodiversity lens strategies to extend to phylogeny-based models. Thus, the phylogenetic beta diversity framework may allow GEO BON to make effective use of cases (as in microbial ecology) where phylogenetic estimates precede taxonomic work. Methods such as ED then can assess losses in phylogenetic diversity. Lens applications of this kind will also take advantage of the phylogenetic estimates, and associated geographic distribution data, produced by emerging large-scale DNA barcoding programs (for discussion, see [57]).

**Figure 6.**
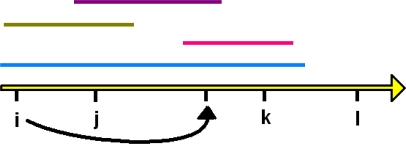
Under the unimodal model, climate change scenarios can be interpreted as causing loss of phylogenetic diversity (evolutionary features) and/or producing new community combinations of features. A hypothetical climate gradient (yellow arrow) shows positions of four localities **i**, **j**, **k**, and **l**. ED calculations indicate changes in communities under climate change scenarios. For details, see text following the figure. Using ED, and its assumption of unimodal response to gradients, we can interpret features as uniformly distributed along the climate gradient, with a uniform distribution of extent-sizes along the gradient. Here, four such features are shown, represented by the blue, red, green and purple line segments (height of segment above the gradient is arbitrary). Suppose climate change implies that locality **i** shifts in position along the climate-related gradient to the new location shown by the arrow. The new community at **i** will consist of a novel combination of features – retaining some existing features (blue), losing some features (green), and (if organisms can disperse to suitable new locations) gaining some features (red and purple) that previously were not found in **i** but were found in other localities. ED calculations (based on combining various ED-complementarity values [51]) indicate the relative numbers of these different feature types.

GEO BON and related programs also may explore scenarios in which human impacts change the values of environmental variables for localities. We have already described ED-based prediction of additional features gained from new sample localities; this kind of calculation naturally extends to the evaluation of existing localities that have changed in environmental values, and so moved to a new position in gradients space. Such a shift in position may be expected under climate change scenarios, when one or more gradients in the environmental space correspond to key climate-related variables. We can predict new phylogenetic community composition under climate change scenarios by using the unimodal features model and ED analyses (Figure 6).

## 7. Conclusions

The phylogenetic beta diversity framework pioneered by microbial ecologists has been successful over a range of studies in revealing environmental gradients that explain turnover in phylogenetic composition. The features-level model presented in this study suggests that this success arises from several key linkages among models and methods. First, the PD-dissimilarities operate as if we are calculating dissimilarities at the level of evolutionary features, because they are compatible with the PD assumption that “shared ancestry accounts for shared features”. Second, because they are Bray-Curtis type dissimilarities, they are robust to variations in unimodal response [12]—and this appears to match the observed relationship between phylogenetic branches/features and gradients space. This phylogenetic unimodal response model where “shared environment accounts for shared features” also suggests general applicability of existing robust methods, including ordination, clustering and ED. These are applicable in a variety of contexts where we have good estimates of phylogeny and its geographic distribution among sample sites. Applications include conservation assessments, search for new commercial products, impacts monitoring, and exploration of drivers of community composition.
